# Aerobic Fitness, B-Vitamins, and Weight Status Are Related to Selective Attention in Children

**DOI:** 10.3390/nu14010201

**Published:** 2021-12-31

**Authors:** Lauren B. Raine, Jennifer N. H. Watrous, Katherine McDonald, Nicole E. Logan, Naiman A. Khan, Arthur F. Kramer, Charles H. Hillman

**Affiliations:** 1 Department of Physical Therapy, Movement, & Rehabilitation Sciences, Northeastern University, Boston, MA 02115, USA; c.hillman@northeastern.edu; 2 Department of Psychology, Northeastern University, Boston, MA 02115, USA; j.hunt@northeastern.edu (J.N.H.W.); mcdonald.kath@northeastern.edu (K.M.); logan.n@northeastern.edu (N.E.L.); a.kramer@northeastern.edu (A.F.K.); 3 Department of Kinesiology and Community Health, University of Illinois at Urbana-Champaign, Urbana, IL 61801, USA; nakhan2@illinois.edu; 4 Beckman Institute, University of Illinois at Urbana-Champaign, Champaign, IL 61801, USA

**Keywords:** cognition, childhood, obesity, fitness, nutrition

## Abstract

There is an increasing prevalence of poor health behaviors during childhood, particularly in terms of physical activity and nutrition. This trend has occurred alongside a growing body of evidence linking these behaviors to cognitive function. B-vitamins are thought to be particularly important in the neural development that occurs during pregnancy, as well as in healthy cognitive aging. However, much less is known regarding the role of B-vitamins during childhood. Given that preadolescent childhood is a critical period for cognitive development, this study investigated the relationship between specific aspects of nutrition, particularly B-vitamins, and related health factors (e.g., body mass, fitness) on selective attention in children. Children (*n* = 85; 8–11 years) completed a selective attention task to assess inhibition. Participant’s dietary intake was collected using the Automated Self-Administered 24-h dietary assessment tool. Correlations between specific nutrients, BMI, fitness, and task performance were investigated. After accounting for demographic variables and total caloric intake, increased B-vitamin intake (i.e., thiamin and folic acid) was associated with shorter reaction times (*p*’s < 0.05), fitness was associated with greater response accuracy (*p* < 0.05), and increased BMI was related to increased variability in reaction times (*p* < 0.05). Together, these findings suggest that aspects of health may have unique contributions on cognitive performance. Proper physical health and nutrition are imperative for effective cognitive functioning in preadolescent children. Targeted efforts aimed at health education amongst this population could ensure proper cognitive development during school-age years, providing a strong foundation throughout life.

## 1. Introduction

Numerous factors are important for optimal child development, in particular proper nutrition and physical activity. Childhood is a critical period for the development of lifelong habits and healthy behaviors [1]. Recently, dietary trends have shifted towards high-density processed foods, with the majority of calories now consumed from calorically dense but nutritionally poor foods [2,3]. In the U.S., less than one in ten children consume the recommended daily amounts of vegetables and only four in ten children eat enough fruit [4]. Poor nutrition quality during childhood is associated with long term adverse health outcomes, including some cancers and high blood pressure among others. In addition to the physical consequences of an unhealthy diet, students with lower diet quality also performed more poorly academically in terms of language and mathematics performance [5]. In addition, many children are already affected by a serious public health concern, as 19.3% of the pediatric population in the U.S. has obesity, including 6.1% with severe obesity and an additional 16.1% with overweight [6,7]. This trend presents complications to physical health and cognition, including scholastic performance [8,9,10,11,12,13,14,15,16,17]. Children with obesity have decreased cognitive performance [18], including poorer attention [8,9], increased impulsivity [10,11], decreased inhibition [12,13,14], decreased cognitive flexibility [15] and worse academic achievement [16,17]. Physical health complications associated with obesity encompass various systems such as: cardiovascular, endocrine, gastrointestinal, respiratory, musculoskeletal, and psychological [19,20,21]. Children with obesity also exhibit different lifestyle behaviors, for example they engage in less physical activity than their normal weight peers [22]. It is currently recommended that children achieve 60 min of moderate to-vigorous physical activity each day to optimize health benefits [23]. Unfortunately, only a quarter of all U.S. children meet the recommended activity guidelines [24,25]. This is concerning because physical activity is associated with a number of positive outcomes including reduced risk of chronic diseases and some cancers [23,26], as well as significant reductions in cardiometabolic disease risks, such as healthy weight maintenance [27], waist circumference, blood pressure, triglycerides, cholesterol, and insulin [26].

Related to physical activity, higher aerobic fitness is also inversely related to cardiovascular and metabolic risk factors [28,29]. Higher fitness during childhood is associated with advantageous cognitive function, with greater benefits observed for aspects of cognitive control. Cognitive control refers to goal-directed behavior involved in perception, memory, and action [30,31]. One of the core cognitive control processes is inhibition [32,33,34]. Inhibition refers to the ability to suppress task irrelevant information in the environment and withhold a prepotent or impulsive response [35]. Previous research indicates that higher fit children outperform their lower fit peers on inhibitory control tasks, with the largest differences in performance observed in the most difficult task conditions [36,37].

Inhibitory control is also implicated in childhood nutrition. After accounting for important variables such as age and IQ, increased dietary fiber intake is associated with greater accuracy on an attentional inhibition task. These findings suggest that during childhood, diet quality influences performance on challenging cognitive tasks [38]. In contrast, children who consume a diet high in saturated fats exhibit slower reaction times, as well as impaired cognitive flexibility [39], a cognitive process used in shifting, selecting attention, and modifying response. Relational memory performance is also poorer with increased consumption of saturated fatty acids and refined sugar during childhood. In contrast, greater intake of omega-3 fatty acids is related to improved relational memory performance [40]. In addition to macronutrients, the role of micronutrients such as vitamins on physical and cognitive health is of growing interest. 

B-vitamins are water soluble, perform essential roles in cellular functioning, and are critical for brain and psychological functions [41]. Much of the work examining the role of vitamins and cognition has taken place at the extremes of the lifespan. Specifically, folate (vitamin B-9) is implicated in brain development through its role in closing the neural tube during fetal development [42]. A deficiency in vitamin B-12 during pregnancy has also been associated with an increased risk of neural tube defects [43,44]. Within the last 25 years, folic acid has been added to enrich foods such as breads, flours, rice, and other grain products [45]. Deficiencies in specific vitamins may impact brain functions, with important implications for cognitive function. 

There is limited and mixed research regarding B-vitamins and cognitive function in children. In Kenya, results from 554 seven-year-old children demonstrate a significant positive relationship between vitamin B-12 and riboflavin with cognitive test scores (digit span), even after controlling for important variables such as energy intake and socio-economic status [46]. Additionally, after 37 days of supplementation with B-vitamins, 101 children in China showed increased performance on a letter selection task [47]. Further, in a large study of U.S. children, higher amounts of folate measured in blood were associated with higher reading and block design scores, while no such association was observed for vitamin B-12 [48]. In contrast, vitamin B-12 concentrations were significantly inversely associated with short-term memory, retrieval ability, and mental processing after controlling for hemoglobin, folate status, and height-for-age Z scores in 598 school children in India [49].

One laboratory-based cognitive task that has been used to examine obesity, physical activity, and nutrition on cognition is an inhibitory control task known as the flanker task. This task allows for interference manipulation in order to modulate inhibitory control demands [50]. The flanker task requires selective attention, or the ability to focus on certain features of the stimulus environment while ignoring others [51,52,53]. 

Current epidemiological evidence indicates that childhood obesity rates have tripled in the last 40 years [54], physical activity levels have dropped, with approximately 75% of children failing to meet the recommended guidelines of at least 60 min of moderate-to-vigorous PA every day [23,55], and consumption of poor dietary patterns is on the rise, such that children consume more ultra processed foods now than 20 years ago [2]. Therefore, research is critically needed to understand the cognitive health implications related to each of these health factors. Thus, the current study examined the independent roles of fitness, obesity, and B-vitamins in a well characterized group of children to better understand the specific role of each factor on inhibitory control. It was hypothesized that healthier behaviors, including increased fitness, decreased BMI, and increased B-vitamins, would relate to better performance on a task of inhibitory control, specifically in the most challenging task conditions. 

## 2. Materials and Methods

### 2.1. Participants 

Participants in the study were between 8–11 years old. Exclusionary criteria included a medical diagnosis of attention deficit disorder or attention deficit hyperactivity disorder, currently taking medications for neurological disorders, specialized education due to educational or attentional disorders, or inability to complete a maximal exercise test. All participants provided written assent and their legal guardians provided written informed consent in accordance with the Institutional Review Board at Northeastern University.

Demographic information for the sample can be found in Table 1. Demographic variables included age, sex, pubertal status, parent education, household income, relative VO_2_ max (kg/mL/min), VO_2_ percentile, BMI (kg/m^2^), and BMI percentile. Parents/guardians completed a measure of pubertal status to ensure prepubescence of participants [56]. Parental education for each participant was bifurcated into two groups, one group had less than an advanced degree and the other group had an advanced degree or greater. Household income was also bifurcated into two groups, greater than or less than $100,000 annual income. The Kaufman Brief Intelligence Test Second Edition (KBIT-2; Kaufman and Kaufman, 2004) is a standardized test (with a mean = 100 ± 15) used to measure generalized intelligence (IQ) [57]. The KBIT-2 is a commercially available paper and pencil-based assessment of cognitive abilities that has been age normed. In the present study, the initial sample consisted of 88 children; however, three children were removed with IQ < 85. Thus, the final sample consisted of 85 children.

### 2.2. ASA24 

The Automated Self-Administered 24-h (ASA24) nutrition recall software was used to measure participants’ dietary intake. One 24-h food recall was completed by parents with input from the child when needed. Prior research has found the ASA24 to be a feasible method for parent-proxy reporting of children’s dietary intake [58]. The ASA24 is a dietary assessment tool developed by the National Cancer Institute (NCI) to collect data regarding dietary intake. An adaptation of the United States Department of Agriculture’s (USDA) Automated Multiple-Pass Method (AMPM) and the Food Intake Recording Software System (FIRSSt), these “food-diaries” include a web-based tool, which records a single day food record and automatically codes the data for use in epidemiological, interventional, behavioral, or clinical research, and education. Key nutrient variables extracted for the present analyses included total energy(kcal) and B-vitamins (i.e., thiamin, riboflavin, niacin, pyridoxine, folate, folic acid, and cobalamin). 

### 2.3. Cardiorespiratory Fitness (VO_2_)

VO_2_max was used as the measure of cardiorespiratory fitness [59]. Prior to the start of the cardiorespiratory fitness test, standing height and weight measurements were taken with children wearing lightweight clothing and no shoes. Height and weight were measured using a Health o meter 500kl digital medical scale (Sunbeam Products, Boca Raton, FL, USA). An indirect calorimetry system (COSMED Quark CPET OMNIA, Concord, California) was used to measure children’s maximal oxygen consumption during a modified Balke protocol [60]. After a brief warmup, children walked/ran at a constant speed on a treadmill with incline increases of 2.5% every 2 min until volitional exhaustion. During the test, children wore a heart rate (HR) monitor across their chest to determine their maximal heart rate. Every two minutes, ratings of perceived exertion (RPE) were assessed using the children’s OMNI Scale [61], which uses a 0–10 pictorial scale to represent perceived physical effort. In addition, children’s feelings were measured every two minutes on the Feeling Scale (FS). Relative peak oxygen consumption was expressed in milliliters of oxygen consumed per kilogram of bodyweight per minute. It was based upon maximal effort defined by a plateau in oxygen uptake corresponding to an increase of less than 2 mL/kg/min despite an increase in exercise workload; or a combination of the following: (1) a maximal HR ≥ 185 beats-per-minute [60], (2) respiratory exchange ratio (RER) ≥ 1.0 [62], and/or (3) RPE ≥ 8 [61]. Finally, based on the child’s age and sex, VO_2_max percentile (VO_2_max%) was determined from normative data [63].

### 2.4. Weight Status Assessment

BMI was calculated as kg/m^2^, using each participant’s weight and height measured in kilograms (kg) and meters (m), respectively. Height and weight were measured using a Health o meter 500 kl digital medical scale (Sunbeam Products, Boca Raton, FL) while participants were barefoot and wearing lightweight clothing. BMI percentile was calculated based on CDC growth charts for children and teens (ages 2–19 years), accounting for age and sex [64].

### 2.5. Inhibition Task 

A modified version of an Eriksen flanker task was used to assess inhibitory control [50]. Participants were presented with a central target amid an array of four irrelevant flanker stimuli, which were congruent or incongruent to the central target stimulus, and asked to distinguish the centrally presented target stimulus from interfering flanking stimuli. Congruent trials consisted of an array of five stimuli facing the same direction (e.g., >>>>>, <<<<<), while incongruent trials consisted of the four flanking stimuli facing the opposite direction of the target (middle) stimulus (e.g., >><>>, <<><<). The incongruent trials require a greater amount of interference control due to perceptual interference raised by the flanking arrows pointing in the opposite direction of the central target arrow, which triggers multiple response mappings. Interference is created by manipulating the congruency of the target and flanking stimuli, such that in the congruent condition, all stimuli engender the same response mapping, whereas in the incongruent condition, the target and flanking stimuli engender alternative response mapping. Since target and flanking stimuli activate opposing action schemas, responses to congruent trials are typically faster and more accurate than incongruent trials [65]. Furthermore, incongruent trials require greater amounts of inhibitory control since target and flanking stimuli activate multiple action schemas [66]. Prior to beginning the task, participants were provided with practice trials to ensure that they understood the task and could perform at a level above 50%. Participants viewed the stimuli on a computer screen positioned focally at a distance of 1 m using E-Prime3 software. Children completed 156 trials, with stimuli presented for a duration of 150 ms and a variable inter-stimulus interval of 1300, 1500 and 1700 ms. The congruent and incongruent trials were equiprobable and were presented in a random sequence. Participants were instructed to respond using a response pad as quickly and accurately as possible with a button press on the side corresponding to the directionality of the central target stimuli amid either congruent or incongruent flanking stimuli. Performance variables were collected for congruent and incongruent trials. Accuracy was calculated as the percentage of correct responses. Mean reaction time (RT) was calculated for correct responses as the time in milliseconds (ms) from stimulus onset until response execution. Standard deviation of reaction time (SDRT) was calculated based on the RT dispersion from the mean. 

### 2.6. Statistical Analysis

Initial Pearson product–moment correlations were conducted between dependent variables from performance on the flanker task and all demographic variables (e.g., age, sex, pubertal status, income, parental education, IQ). Any variable that significantly correlated with the dependent variable was included as a covariate in the first step of the multiple linear regression analyses. Next, separate multiple hierarchical linear regression analyses were conducted for each performance variable on the flanker task. In Step 1, the dependent variables were regressed on all significantly correlated demographic variables (e.g., pubertal status, income, parental education, IQ). If no demographic variable was significantly correlated with the outcome, this step was skipped. To determine the unique contribution of each independent variable, the final step included nutrient variables, fitness and BMI, which were independently entered into Step 2. The change in R^2^ values between the two steps was used to judge the independent contribution of these measures for explaining the variance in the dependent variables of interest beyond that of demographic variables.

To adjust for overall energy intake, vitamin intake was normalized to intake per 1000 kcal within participants before analyses. Statistics were performed using SPSS 27 (IBM, Somers, NY, USA).

## 3. Results

Demographic information for the sample can be found in Table 1.

### 3.1. Correlations

Correlations between flanker variables and demographics are presented in Table 2. Age was most frequently correlated with flanker performance variables, such that older children exhibited superior flanker performance (see Table 2). 

### 3.2. Accuracy

#### 3.2.1. Congruent Accuracy

For congruent trials, the Step 1 regression analysis was significant, adjusted R^2^ = 0.05, F(1, 83) = 4.37, *p* = 0.04. In Step 2, the addition of BMI, fitness, or B-vitamins did not account for an incremental amount of variance in accuracy beyond associated descriptive variables (see Table 3).

#### 3.2.2. Incongruent Accuracy

For incongruent trials, the Step 1 regression analysis was significant, adjusted R^2^ = 0.14, *F*(2, 82) = 6.392, *p* = 0.003. With the addition of VO_2_max, Step 2 was also significant, ΔR^2^ = 0.05, *F(*3, 81) = 6.06, *p* = 0.001, such that greater fitness was associated with greater incongruent accuracy, with VO_2_max accounting for an incremental amount of variance in incongruent accuracy beyond the associated descriptive variables, β = 0.22, *t*(82) = 2.20, *p =* 0.03. The addition of BMI and the B-vitamins did not account for an incremental amount of variance in accuracy beyond the associated descriptive variables (see Table 4).

### 3.3. Mean RT

#### 3.3.1. Congruent Mean RT

For congruent trials, the Step 1 regression analysis was significant, adjusted R^2^ = 0.10, *F*(1, 83) = 9.14, *p* = 0.003. With the addition of folic acid, Step 2 was also significant, ΔR^2^ = 0.08, *F(*2, 82) = 8.71, *p* ≤ 0.001, such that greater dietary folic acid was associated with shorter congruent RT, with dietary folic acid accounting for an incremental amount of variance in congruent RT beyond the associated descriptive variables, β = −0.28, *t*(82) = −2.75, *p =* 0.007. Separately, the addition of thiamin in Step 2 was also significant, ΔR^2^=0.05, *F(*2, 82) = 7.65, *p* = 0.001, such that greater dietary thiamin was associated with shorter congruent RT, with dietary thiamin accounting for an incremental amount of variance in congruent RT beyond the associated descriptive variables, β = −0.24, *t*(82) = −2.38, *p =* 0.02. Finally, the addition of VO_2_max in Step 2 was also significant, ΔR^2^ = 0.05, *F(*2, 82) = 7.41, *p* = 0.001, such that greater fitness was associated with longer congruent RT, with VO_2_max accounting for an incremental amount of variance in congruent RT beyond the associated descriptive variables, β = 0.22, *t*(82) = 2.28, *p =* 0.03. The addition of BMI and the remaining B-vitamins did not account for an incremental amount of variance in accuracy beyond associated descriptive variables (see Table 5). 

#### 3.3.2. Incongruent Mean RT

For incongruent trials, the Step 1 regression analysis was significant, adjusted R^2^ = 0.06, *F*(1, 83) = 5.30, *p* = 0.02. With the addition of folic acid, Step 2 was significant, ΔR^2^ = 0.08, *F(*2, 82) = 6.61, *p* = 0.002, such that greater dietary folic acid was associated with shorter incongruent RT, with dietary folic acid accounting for an incremental amount of variance in incongruent RT beyond the associated descriptive variables, β = −0.28, *t*(82) = −2.74, *p =* 0.008. The addition of thiamin in Step 2 was also significant, ΔR^2^ = 0.08, *F(*2, 82) = 6.49, *p* = 0.002, such that greater dietary thiamin was associated with shorter incongruent RT, with dietary thiamin accounting for an incremental amount of variance in incongruent RT beyond the associated descriptive variables, β = −0.28, *t*(82) = −2.67, *p =* 0.008. The addition of VO_2_max, BMI and the remaining B-vitamins did not account for an incremental amount of variance in accuracy beyond associated descriptive variables (see Table 6).

### 3.4. SDRT

#### 3.4.1. Congruent SDRT

For congruent trials, the Step 1 regression analysis was significant, adjusted R^2^ = 0.11, *F*(1, 83) = 19.05, *p* ≤ 0.001. With the addition of BMI, Step 2 was also significant, ΔR^2^ = 0.07, *F(*2, 82) = 14.29, *p* ≤ 0.001, such that greater BMI was associated with greater variability in RT, with BMI accounting for an incremental amount of variance in congruent SDRT beyond the associated descriptive variables, β = 0.28, *t*(82) = 2.87, *p =* 0.005. The addition of VO_2_max and the B-vitamins did not account for an incremental amount of variance in accuracy beyond the associated descriptive variables (see Table 7).

#### 3.4.2. Incongruent SDRT

For incongruent trials, the Step 1 regression analysis was significant, adjusted R^2^ = 0.19, F(1, 83) = 19.38, *p* ≤ 0.001. With the addition of BMI, Step 2 was marginally significant, ΔR^2^ = 0.04, F(2, 82) = 11.88, *p* ≤ 0.06, such that greater BMI was associated with greater SDRT, with BMI accounting for a marginal incremental amount of variance in incongruent RT variability beyond the associated descriptive variables, β = 0.19, *t*(82) = 1.93, *p* = 0.006. The addition of VO_2_max and the B-vitamins did not account for an incremental amount of variance in accuracy beyond associated descriptive variables (see Table 8).

## 4. Discussion

This investigation assessed the independent effects of B-vitamin intake, aerobic fitness, and BMI using a cognitive task that manipulated inhibitory control demands. The findings from the current study complement previous research within the field via examination of behavioral outcomes in response to a selective attention task in preadolescent children in relation to a variety of health factors, including nutrition, aerobic fitness, and body composition. The merging of these health factors affords a unique opportunity to examine the specific, nuanced roles of each. Collectively, these findings provide evidence that greater intake of folic acid and thiamin, higher aerobic fitness, and lower BMI during childhood is associated with greater response accuracy, shorter RT, and less response variability during performance of an inhibitory control task that requires selective attention.

Novel to this investigation was the multifaceted analyses involving various measures of health to better understand the specificity of each on task performance. In general, higher fitness was associated with greater response accuracy in the task condition requiring greater amounts of inhibitory control (i.e., incongruent task condition). Increased BMI was associated with greater response variability. Finally, increased dietary thiamin and folic acid were related to faster responses across both conditions of the task, suggesting generalized benefits to tasks that modulate inhibitory control demand. These findings highlight the specific relations that each measure of health may have on cognition in preadolescent childhood. The results of this investigation are significant due to the nationwide increase in levels of obesity [6], declines in PA among children [67], and a growing lack of adequate sources of good nutrition [4]. Taken together, these findings suggest that future studies that modify lifestyle behaviors may have the potential improve cognition, particularly selective attention and inhibition. 

One relevant lifestyle behavior is nutrition, specifically B-vitamins, as they are required for essential brain metabolic pathways and are vital for brain development and maintenance [68,69]. B-vitamins are important for methylation, particularly during cell repair. Further, B-vitamins are closely linked to the physiological metabolism of homocysteine, such that low levels of certain B-vitamins result in dysregulated and elevated homocysteine levels, which increase the risk for cognitive impairment in older adults [70,71]. B-vitamins appear beneficial for a variety of populations, such as adults over 40 years old and individuals with mild cognitive impairment, in terms of global cognitive function and especially for tasks of episodic memory [72]. Previous studies in older adults have linked decreased folate and thiamine with cognitive impairment and neurodegeneration [73,74]. Thiamine has an important role in energy releasing reactions in the body and is necessary for proper nervous system functioning. Food sources of thiamin include whole grains, meat, and fish [75]. Thiamine deficiency produces selective cell death in the brain and a loss of neurons, which have been linked to cognitive deficits. In institutionalized older adults, thiamine deficiency is associated with higher levels of depression and Alzheimer’s disease [73]. Interestingly, thiamine deficient rats have a longer response time to an electric stimulus [76] and impaired cognition [77], as evidenced by impaired performance on learning, avoidance [78,79], and water maze tasks [80,81]. The present findings are in agreement, such that increased dietary thiamine was related to faster response times on a task of inhibition and selective attention. 

Another important B-vitamin is B-9 (folate) and includes the various forms of the vitamin including folic acid, dihydrofolate, tetrahydrofolate, 5, 10-methylenetetrahydrofolate and 5-methyltetrahydrofolate [82]. Although the terms “folate” and “folic acid” are often used interchangeably, folic acid is an oxidized synthetic form of folate and is used to fortify foods and has been shown to prevent against neural tube defects [83,84]. Serum folate was positively associated with verbal fluency, memory recall, and letter search performance, which involves sustained attention and processing speed in a sample of 4,166 older adults in the European HAPIEE study [85]. Over a five year monitoring period, women who consumed folate levels below the recommended intake had an increased risk of mild cognitive impairment and dementia [86]. In older adults with mild cognitive impairment, supplementation with folic acid and other B-vitamins slowed the rate of brain atrophy [87]. Interestingly, our findings revealed a positive influence of consumption of folic acid, but not folate, on reaction time during both congruent and incongruent trials. As mentioned earlier, both folate and folic acid specifically have been shown to be beneficial for cognition and brain in other studies. However, one of the implications of the findings from the present work is that food sources that are fortified with folic acid, compared to foods with naturally occurring folate, may have contributed to the greater attentional and inhibitory control abilities in our sample. Alternatively, folic acid might be the largest constituent of folate consumed in the diet in the current sample. Nevertheless, our findings add to this body of literature and suggest that increased dietary folic acid is related to faster response times in children during tasks that manipulate inhibitory control and selective attention demand.

During preadolescent childhood, selective attention is crucial since it involves prolonged attention and control of one’s actions [21,22], which are germane to scholastic success. Findings from the present study are in agreement with previously published data, indicating that lower fitness may relate to general impairments in cognitive control and overall brain health [88,89,90,91]. Specifically, increased aerobic fitness was related to improved performance on the task conditions requiring an upregulation of inhibitory control (i.e., the incongruent trials), suggesting that fitness may have a specific benefit to this aspect of cognition. The associations between congruent RT and fitness oppose the previously published literature, such that the present investigation found that higher fitness was related to longer response times on trials with lower inhibitory demands. There are a few possible reasons for this finding. For example, the sample was predominately lower fit and this reduced fitness range may have impacted results by not accounting for the breadth of childhood fitness. It should be further noted that data collection was impacted by the COVID-19 pandemic, which may have related to several unmeasured physical and mental health factors. 

With increased BMI, children often display poorer inhibition [12]. That is, children with obesity perform more poorly on tests of inhibitory control as evidenced by longer RT across a variety of inhibitory control tasks, including the flanker [12] and Stroop [16] tasks. Such findings suggest that with higher BMI, individuals become less able to modulate inhibitory control to meet the increased task demands [15]. In the present study, these effects extend to deficits in intraindividual response variability, as measured via SDRT. SDRT reflects the within-person fluctuations in response time. These within person fluctuations in behavioral performance are a useful tool for differentiating performance during tasks requiring variable amounts of interference control. Previous research suggests that greater variability in mean RT has been observed in children relative to young adults [92,93] and in children with attention-deficit and hyperactivity disorder [94]. Prior research suggests that lower fit children exhibit more variable performance relative to their higher fit peers, as indicated by greater SDRT [88,95,96]. 

In concert with the present study, previous research in children also found that greater adiposity is related to greater with-in person variability, highlighting the negative influence that excess adiposity may already exert during childhood. The current findings are in agreement and suggest an association between higher BMI and increased SDRT, which reflects more variable responses across trials. In contrast, decreases in SDRT are related to white matter tract maturation and increased functional connectivity, suggesting a neural substrate for observed differences in variability of task performance [97]. Thus, the present findings suggest that greater BMI may be a marker of decreased or delayed neural maturation during preadolescent development, including maturation of white matter integrity. Increased BMI in preadolescent childhood is also associated with lower grey and white matter volume in brain regions implicated in cognitive control and learning, suggesting an association between increased BMI and reduced cognitive outcomes [98,99,100,101]. The causal direction of the association between adiposity and cognition remains unknown; however, it could be that poorer inhibition precedes increases in BMI and may predispose a child to unhealthy behaviors that subsequently increase BMI, as research suggests that inhibitory control ability can predict weight status two and a half years later [102]. 

While this study adds to a growing body of literature, it is not without limitations. For example, there may be unmeasured or unobserved factors such as cognitive stimulation or parenting that are important and could confound the associations between nutrition, body composition, fitness, and cognition. These factors were not measured in the current investigation, but are important to consider in future studies. The study was cross-sectional and relies on dietary recall and, thus, does not represent long-term dietary status. However, the ASA24 is a widely used tool in nutrition research. Findings from prior studies suggest that parents are able to accurately report what their children ate and drank the day prior [103]. Since 2009, when the ASA24 was released, more than 6000 studies have registered to use ASA24 and more than 521,000 recall or record days have been collected as of January 2020 (National Cancer Institute). ASA24-2020 is the version used during the collection of dietary data for this research. Future studies should include either long term measures and/or interventions to confirm that increased dietary B-vitamins are related to improved cognitive performance. Future studies should also investigate serum levels of B-vitamins to determine if the consumed levels and the levels in the blood are both related to cognitive performance. In addition, all of the children included in this study had normal to above normal IQ and the education level of parents was high. Thus, additional research with larger samples is needed to determine if these findings extend to other groups of children.

Today’s children fail to meet the federal guidelines for healthy diets, with scores nearly half of what is recommended. This puts individuals at risk of diet related adult chronic diseases [104]. In addition, children are becoming increasingly unfit and inactive, with lower fitness and higher BMI associated with increased cardiovascular risk [105], as well as decreased brain health, which have implications for cognitive and scholastic performance [36,37,88,89,90,106,107]. Accordingly, findings from this study add to a growing body of research indicating the beneficial relation of health factors on cognitive control and specifically selective attention and inhibitory control, in preadolescent children. Furthermore, research suggests that changes in physical activity and diet are beneficial in terms of preventing and treating childhood obesity [108] and the present findings add to this benefit and suggest benefits also extend to cognition with healthy behaviors. Given that physical inactivity, obesity, and poor nutrition are major public health concerns with a myriad of health consequences [82,83], this investigation provides critical evidence for the specific roles of different health factors on cognitive health in children. Such findings have implications for the educational environment, physical health, and brain health of today’s children.

## Figures and Tables

**Table 1:** Participant demographic information (Mean ± SE).

Characteristic	
N, % female	85, 44%
Age (years)	9.93 ± 0.08
Household Income	Less than $100,000/year = 38% $100,000 or more/year = 62%
Highest Level of Mother’s Education	Less than advanced degree = 45% Advanced degree or more = 55%
Highest Level of Father’s Education	Less than advanced degree = 53% Advanced degree or more = 47%
IQ	115.23 ± 1.47
Pubertal Status	1.46 ± 0.06
VO_2_ relative (kg/mL/min)	43.44 ± 0.77
VO_2_ percentile	31.81 ± 3.28
BMI (kg/m^2^)	17.94 ± 0.34
BMI%	56.34 ± 3.36 (27% overweight or obese)
Congruent Accuracy (% correct)	81.90 ± 1.41
Incongruent Accuracy (% correct)	66.28 ± 1.92
Accuracy Interference (% correct)	15.63 ± 1.54
Congruent RT (ms)	563.52 ± 9.47
Incongruent RT (ms)	627.11 ± 11.23
RT Interference (ms)	63.58 ± 5.25
Congruent SDRT	140.51 ± 4.49
Incongruent SDRT	158.05 ± 5.95
SDRT Interference	17.54 ± 4.01
Energy (kcal)	1774.47 ± 64.80
Thiamin (B-1) (mg)	1.49 ± 0.06
Riboflavin (B-2) (mg)	1.76 ± 0.09
Niacin (B-3) (mg)	19.32 ± 1.17
Pyridoxine (B-6) (mg)	1.64 ± 0.11
Folate (mcg)	378.96 ± 20.44
Folic Acid (mcg)	202.42 ± 17.81
Cobalamin (B-12) (mcg)	4.25 ± 0.29

Note: IQ = generalized intelligence; SES = socioeconomic status; VO_2_ = maximal oxygen volume.

**Table 2:** Pearson correlations between flanker performance and demographic variables.

	Age	Sex	Pubertal Timing	IQ	Mother Education	Father Education	Household Income
Congruent Accuracy	0.22 *	0.10	−0.03	0.20	0.13	0.17	0.01
Incongruent Accuracy	0.26 *	0.17	−0.13	0.22 *	0.09	0.17	0.02
Accuracy Interference	−0.12	−0.12	0.13	−0.10	0.02	−0.05	−0.01
Congruent RT	−0.32 **	−0.17	−0.17	−0.02	0.05	−0.10	0.01
Incongruent RT	−0.25 *	−0.21	−0.13	0.02	0.06	−0.07	−0.03
RT Interference	0.04	−0.16	0.03	0.09	0.04	0.02	−0.09
Congruent SDRT	−0.43 **	0.001	−0.01	−0.08	−0.10	−0.18	−0.12
Incongruent SDRT	−0.43 **	−0.05	−0.004	0.05	0.03	−0.16	0.02
SDRT Interference	−0.16	−0.07	0.01	0.016	0.15	−0.03	0.16

** Correlation is significant at the 0.01 level; * Correlation is significant at the 0.05 level.

**Table 3:** Congruent Accuracy Regressions.

Congruent Accuracy		ANOVA *F*	ANOVA *P*	∆R^2^	t	β
Step1	Age	4.37	0.04	0.05 *	2.09	0.224
Step 2	BMI	2.31	0.11	0.003	0.54	0.06
Step 2	VO_2_ max	2.44	0.09	0.01	0.73	0.08
Step 2	Thiamin (B-1)	2.47	0.09	0.01	0.77	0.08
Step 2	Riboflavin (B-2)	2.70	0.07	0.01	1.02	0.11
Step 2	Niacin (B-3)	2.65	0.08	0.01	−0.97	−0.10
Step 2	Pyridoxine (B-6)	2.53	0.09	0.01	−0.84	−0.09
Step 2	Folate	2.64	0.08	0.01	0.96	0.10
Step 2	Folic Acid	2.24	0.11	0.002	0.40	0.04
Step 2	Cobalamin (B-12)	2.16	0.12	0.00	0.10	0.01

* ∆R^2^ is significant at *p* < 0.05 level.

**Table 4:** Incongruent Accuracy Regressions.

Incongruent Accuracy		ANOVA *F*	ANOVA *P*	∆R^2^	t	β
Step1	Age	6.392	0.003	0.14 *	2.83	0.29
	IQ				2.49	0.26
Step 2	BMI	4.24	0.008	0.001	−0.26	−0.03
Step 2	VO_2_ max	6.06	0.001	0.05 *	2.20	0.22
Step 2	Thiamin (B-1)	4.52	0.006	0.01	0.89	0.09
Step 2	Riboflavin (B-2)	4.34	0.007	0.004	0.59	0.06
Step 2	Niacin (B-3)	4.23	0.008	0.001	−0.25	−0.03
Step 2	Pyridoxine (B-6)	4.33	0.007	0.003	−0.56	−0.06
Step 2	Folate FOLA	4.33	0.005	0.01	0.92	0.10
Step 2	Folic Acid FA	4.21	0.008	0.000	−0.10	−0.01
Step 2	Cobalamin (B-12)	4.26	0.008	0.001	−0.36	−0.04

* ∆R^2^ is significant at *p* < 0.05.

**Table 5:** Congruent Reaction Time Regressions.

Congruent RT		ANOVA *F*	ANOVA *P*	∆R^2^	t	β
Step1	Age	9.14	0.003	0.10 *	−3.02	−0.23
Step 2	BMI	5.21	0.007	0.01	−1.12	−0.12
Step 2	VO_2_ max	7.41	0.001	0.05 *	2.28	0.22
Step 2	Thiamin (B-1)	7.66	0.001	0.06 *	−2.38	−0.30
Step 2	Riboflavin (B-2)	5.19	0.008	0.01	−1.10	−0.11
Step 2	Niacin (B-3)	5.24	0.007	0.01	−1.14	−0.12
Step 2	Pyridoxine (B-6)	4.75	0.01	0.005	−0.65	−0.07
Step 2	Folate	5.81	0.004	0.03	−1.53	−0.16
Step 2	Folic Acid	8.71	0.00	0.08 *	−2.75	−0.28
Step 2	Cobalamin (B-12)	4.86	0.01	0.01	−0.79	−0.08

* ∆R^2^ is significant at *p* < 0.05.

**Table 6:** Incongruent Reaction Time Regressions.

Incongruent RT		ANOVA *F*	ANOVA *P*	∆R^2^	t	β
Step1	Age	5.30	0.02	0.06 *	−2.30	−0.24
Step 2	BMI	2.71	0.07	0.002	−0.42	−0.05
Step 2	VO_2_ max	4.09	0.02	0.03	1.66	0.18
Step 2	Thiamin (B-1)	6.50	0.002	0.08 *	−2.70	−0.28
Step 2	Riboflavin (B-2)	3.42	0.04	0.02	−1.22	−0.13
Step 2	Niacin (B-3)	4.36	0.02	0.04	−1.81	−0.19
Step 2	Pyridoxine (B-6)	4.25	0.02	0.03	−1.75	−0.18
Step 2	Folate	3.71	0.03	0.02	−1.43	−0.15
Step 2	Folic Acid	6.61	0.002	0.08 *	−2.74	−0.28
Step 2	Cobalamin (B-12)	3.49	0.04	0.2	−1.28	−0.14

* ∆R^2^ is significant at *p* < 0.05.

**Table 7:** Congruent Standard Deviation of Reaction Time Regressions.

Congruent SDRT		ANOVA *F*	ANOVA *P*	∆R^2^	t	β
Step1	Age	19.05	0.000	0.19 *	−4.36	−0.43
Step 2	BMI	14.49	0.000	0.07 *	2.87	0.28
Step 2	VO_2_ max	10.77	0.000	0.021	−1.48	−0.15
Step 2	Thiamin (B-1)	9.88	0.000	0.007	0.87	−0.09
Step 2	Riboflavin (B-2)	9.73	0.000	0.005	0.72	0.07
Step 2	Niacin (B-3)	9.49	0.000	0.001	0.35	0.04
Step 2	Pyridoxine (B-6)	10.29	0.000	0.01	1.20	0.12
Step 2	Folate	10.09	0.000	0.01	−1.05	−0.10
Step 2	Folic Acid	9.84	0.000	0.007	−0.84	−0.08
Step 2	Cobalamin (B-12)	10.17	0.000	0.01	1.11	0.11

* ∆R^2^ is significant at *p* < 0.05.

**Table 8:** Incongruent Standard Deviation of Reaction Time Regressions.

Incongruent SDRT		ANOVA *F*	ANOVA *P*	∆R^2^	t	β
Step 1	Age	19.38	0.000	0.19 *	−4.40	−0.44
Step 2	BMI	11.88	0.000	0.035 †	1.93	0.19
Step 2	VO_2_ max	11.61	0.000	0.03	−1.82	−0.18
Step 2	Thiamin (B-1)	9.67	0.000	0.001	−0.38	−0.04
Step 2	Riboflavin (B-2)	9.56	0.000	0.000	0.14	0.01
Step 2	Niacin (B-3)	9.58	0.000	0.000	0.09	0.01
Step 2	Pyridoxine (B-6)	9.58	0.000	0.000	−0.02	−0.002
Step 2	Folate	11.35	0.000	0.03	−1.69	−0.17
Step 2	Folic Acid	10.45	0.000	0.014	−1.19	−0.12
Step 2	Cobalamin (B-12)	9.66	0.000	0.001	0.37	0.04

* ∆R^2^ is significant at *p* < 0.05; † ∆R^2^ is marginally significant at *p* = 0.06.

## Data Availability

The data presented in this study are available on request from the corresponding author. The data are not publicly available at this time as primary internal analyses are ongoing and in an effort to maintain participant confidentiality.
